# Cemento-osseous dysplasia with a *NOTCH4* mutation: a case report

**DOI:** 10.1007/s00428-025-04212-4

**Published:** 2025-08-15

**Authors:** Gerben E. Breimer, Nard G. Janssen, Anne M. L. Jansen, Pieter J. Slootweg

**Affiliations:** 1 Department of Pathology, University Medical Center Utrecht, Utrecht, The Netherlands; 2 Department of Oral and Maxillofacial Pediatric Surgery, University Medical Center Utrecht, Utrecht, The Netherlands; 3 Department of Pathology, Radboud University Medical Centre, Nijmegen, The Netherlands

**Keywords:** Cemento-Osseous Dysplasia, Fibro-osseous lesions, *NOTCH4* mutation

## Abstract

Cemento-osseous dysplasia (COD) is a benign fibro-osseous lesion of the jaw that can mimic other entities, particularly cemento-ossifying fibroma (COF), both radiologically and histologically. Although recent research has implicated mutations in the RAS-MAPK pathway in COD, its broader molecular landscape remains insufficiently defined. We report the case of a 32-year-old woman with an incidental lesion in the right mandibular angle. Radiographic assessment revealed a poorly demarcated lesion, and histopathological analysis confirmed features consistent with COD, including fibro-osseous tissue with interconnected bony trabeculae lacking osteoblastic rimming. Next-generation sequencing (TSO500 panel) identified a previously unreported *NOTCH4* mutation. This finding expands the spectrum of genetic alterations associated with COD and raises the possibility of Notch signaling involvement in its pathogenesis. Incorporating molecular profiling into the diagnostic workflow may improve discrimination between COD and COF and deepen our understanding of fibro-osseous lesions of the jaw.

## Introduction

Fibro-osseous lesions of the jaw pose diagnostic challenges due to their rarity and overlapping histopathological and radiological features, requiring combined expertise in pathology, radiology, and clinical evaluation for accurate classification [1]. One important entity within this group is cemento-osseous dysplasia (COD), a benign lesion in the tooth-bearing areas of the jaws. It is classified into four subtypes, i.e., periapical, focal, florid, or familial florid cemento-osseous dysplasia subtype, based on the location and distribution of the lesions. COD must be distinguished from cemento-ossifying fibroma (COF) [2]. A key distinguishing feature lies in radiological assessment, particularly the nature of the lesion’s interface with adjacent bone. A sharply demarcated lesion favors the diagnosis of COF, whereas a more diffuse boundary is suggestive of COD.

In recent years, molecular analyses have substantially advanced our understanding and classification of fibro-osseous tumors of the jaw by identifying distinct genetic alterations. For instance, identifying *SATB2* rearrangements appears to be highly specific for psammomatoid ossifying fibroma, facilitating its distinction from histologically overlapping lesions [3]. Additionally, DNA methylation-based approaches have emerged as promising diagnostic tools capable of refining the classification of these complex tumors [4]. Haefliger et al. recently identified activation of the RAS-MAPK pathway in a subset of cemento-osseous dysplasia (COD) cases, highlighting an important molecular aspect of COD pathogenesis [5]. Nevertheless, significant gaps remain in our understanding of the genetic landscape and molecular drivers underlying COD.

In this report, we describe a case of COD harboring a *NOTCH4* mutation, thereby expanding the molecular spectrum associated with this lesion and identifying potential avenues for further investigation into its molecular basis.

## Case

A 32-year-old woman was referred to the maxillofacial surgeon by the orthodontist due to a progressive asymmetry of the mandible, which was suspected to be due to unilateral condylar hyperplasia. In order to assess the eventuality of persistent unilateral condylar growth, a SPECT/CT scan was performed. As a coincidental finding, a circumscript, 10 mm large, round metabolic hotspot was found in the right mandibular angle, exactly in the region where a wisdom tooth had been removed approximately ten years ago. Under local anesthesia, the region was explored and enucleated. A granulomatous tumor was sent for histopathological examination.

## Imaging, histology, and molecular findings

**Fig. 1:**
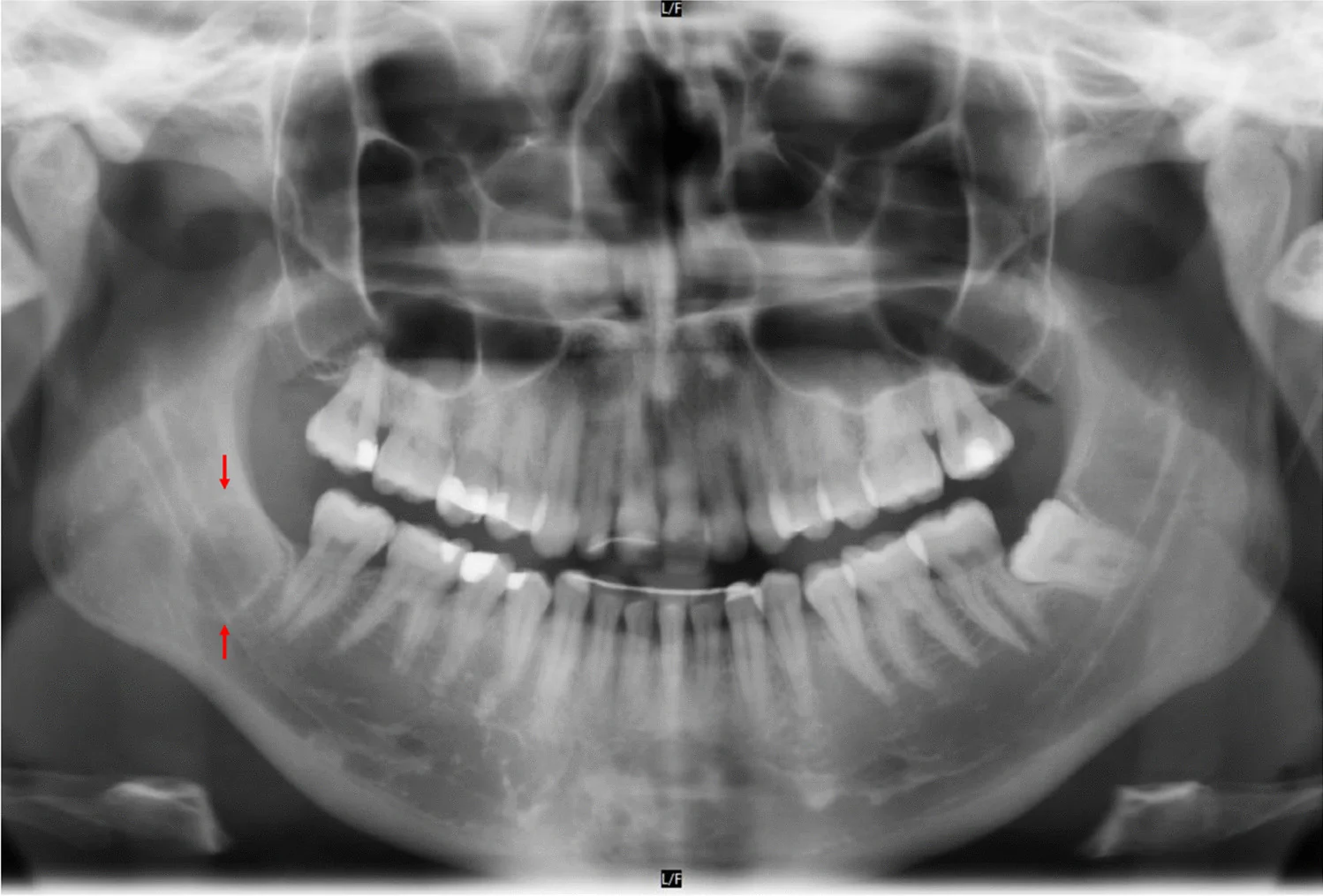
Panoramic X-ray showing a circumscript radio-opacity distally of the right lower second molar (red arrows indicate the lesion and its extent)

**Fig. 2:**
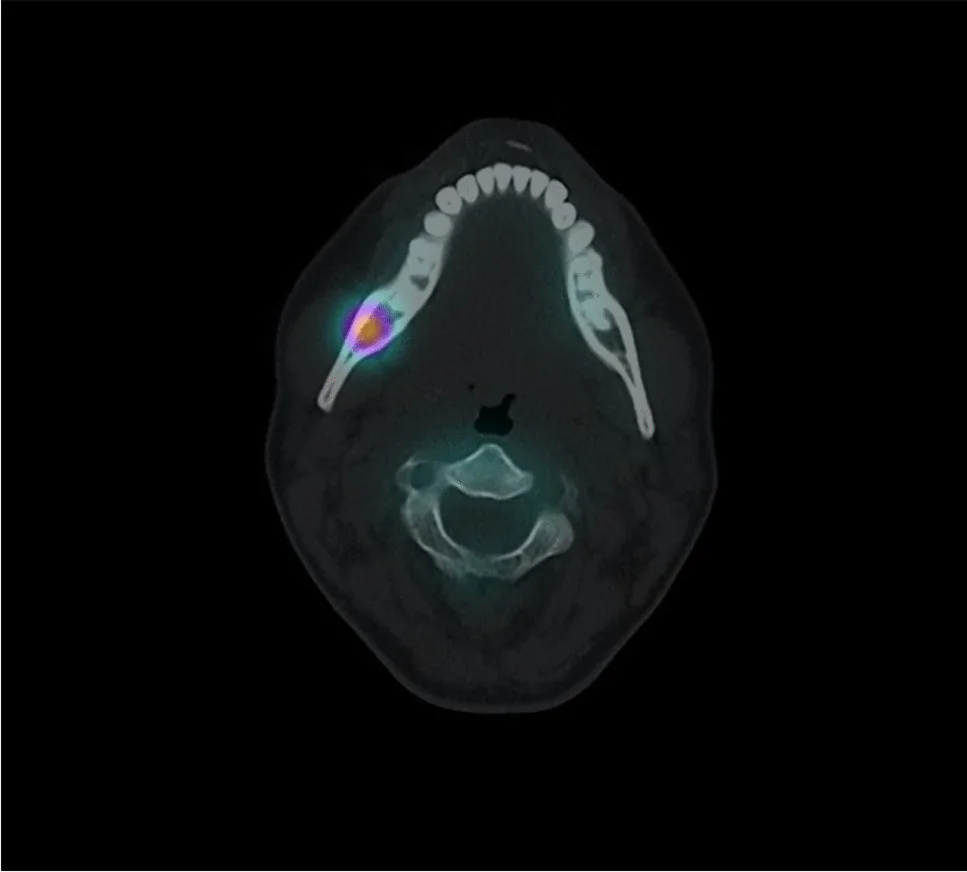
Fused SPECT/CT image showing metabolic activity in the region of the radio-opacity

**Fig. 3:**
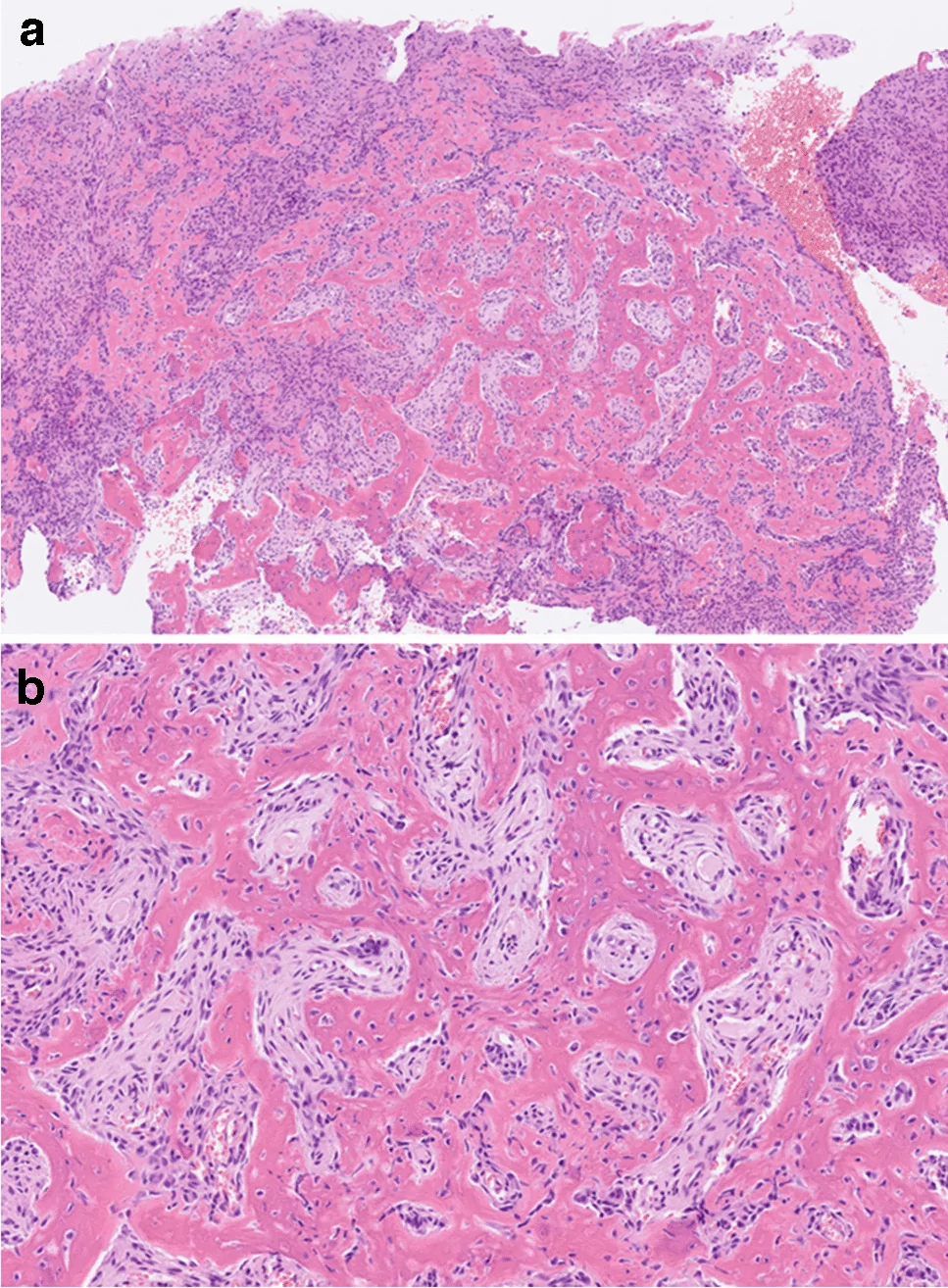
Hematoxylin and eosin-stained micrographs showing a network-like arrangement of interconnected bony trabeculae. (**a**) Low-power overview demonstrating the general architectural pattern, with irregularly anastomosing trabeculae. (**b**) Higher magnification highlights the lack of prominent osteoblastic rimming along the trabecular surfaces

A decisive feature for differentiating COD from COF is the character of the interface between the lesion and adjacent bone: sharp in OF and diffuse in COD. In the orthopantomogram (Fig. 1), no distinct demarcation is observed, favoring a diagnosis of COD. The SPECT/CT shows metabolic activity in the region of the radio-opacity (Fig. 2). There is also a network-like arrangement of interconnected bony trabeculae without a well-defined rim of osteoblasts (Fig. 3), a feature that is uncommon in COF but more frequently observed in COD.

DNA extraction was performed using the Maxwell RSC DNA FFPE Kit (Promega) following the manufacturer’s instructions. Library preparation was performed following the TruSight Oncology 500 High Throughput (TSO500 HT) protocol, and sequencing was carried out on the NovaSeq X + platform (Illumina). Variant calling was done using the Franklin platform (Genoox), and variants were classified according to the five-tiered ACMG guidelines (ranging from benign to pathogenic) [6]. For *NOTCH4* annotation, NM_004557 was used. Splice predictions were done using Alamut VisualPlus (Sophia Genetics) and SpliceAI [7].

Using the TSO500 assay, a splice site variant was identified in intron 12 of the *NOTCH4* gene (c.2021 + 1G > A). This variant has not been previously reported in public databases or the literature but affects the highly conserved canonical splice donor site. In silico analysis predicts complete disruption of normal splicing at this site, supporting classification as a likely pathogenic variant. Loss of this splice site is expected to result in aberrant splicing, likely leading to exon skipping, a frameshift, and subsequent introduction of a premature stop codon—consistent with a loss-of-function (LoF) mechanism.

## Discussion

This case report describes a COD lesion harboring a *NOTCH4* mutation. While the histomorphological features of COD are well established, its molecular pathogenesis has only recently begun to be elucidated. In a recent study of 18 COD cases, somatic mutations were identified in 5 cases (28%), involving genes in the RAS-MAPK signaling pathway, including *BRAF*, *HRAS*, *KRAS*, *NRAS*, and *FGFR3* [5]. It is noteworthy that their sequencing panel did not cover *NOTCH*.

In contrast, another study of 12 COF cases reported no recurrent gene fusions (in 5 cases) or recurrent pathogenic variants, specifically not in the *NOTCH* gene family (using whole-exome sequencing in 11 cases) [8]. However, copy number gains involving chromosome 12 were identified in 3 out of 11 cases (27%) [8]. Additionally, other fibro-osseous lesions demonstrate distinct molecular signatures. For example, psammomatoid ossifying fibroma is associated with *SATB2* rearrangements, while fibrous dysplasia is characterized by *GNAS* mutations [1, 3].

While COD and COF can show overlapping histological features, they are considered distinct clinicopathological entities, with COD being a non-neoplastic, self-limiting lesion and COF a benign, expansile neoplasm. The Notch signaling pathway, implicated in our case via a *NOTCH4* mutation, has previously been described in COF, implying there might be an overlap in molecular patterns in COD and COF [9]. Nevertheless, a common genetic marker does not negate a distinction made on other grounds. Also, a recent DNA methylation-based study by Kleijn et al. reported co-clustering of COD, COF, and juvenile trabecular ossifying fibroma, suggesting some degree of epigenetic similarity [4]. However, the authors caution that this finding likely reflects methodological limitations, such as the lack of lesion-specific molecular markers and the limited number of well-preserved COD and JTOF samples, rather than true biological overlap.

Splice site mutations in Notch family genes, including *NOTCH1* and *NOTCH2*, have been shown to result in LoF and contribute to tumor suppression, for example, in various squamous cell carcinomas [10]. While functional studies for this specific *NOTCH4* variant are lacking, canonical splice site variants are frequently deleterious and result in truncated, non-functional protein products.

Notch4 is a transmembrane receptor that functions within the Notch signaling pathway, which is critical for regulating stem cell maintenance, cell fate determination, differentiation, proliferation, and apoptosis [11]. Although Notch signaling was initially characterized as oncogenic in T-cell leukemia, subsequent studies have demonstrated that its role is context-dependent, with evidence supporting both oncogenic and tumor-suppressive functions depending on the cellular and tissue environment [11]. Notch signaling plays an important role during bone development, bone remodeling, and regeneration, acting as a suppressor of intramembranous bone formation and an inhibitor of osteogenesis [12–14]. Mutations in the *NOTCH* gene family have been implicated in syndromic diseases with skeletal involvement, such as Adams–Oliver syndrome (*NOTCH1*), Alagille syndrome (*NOTCH2*), and Hajdu-Cheney syndrome (*NOTCH2*) [13, 15]. Although the role of *NOTCH4* in benign fibro-osseous lesions has not been previously described, its involvement in this COD case suggests a possible novel mechanism of pathogenesis that warrants further investigation.

Until molecular characteristics are more clearly defined, radiographic imaging remains the primary diagnostic modality for distinguishing COD from COF. Accurate diagnosis is essential, as COD typically follows a conservative, non-interventional management strategy, whereas COF may require surgical removal [5].
